# Editorial: Exercise and type 2 diabetes: reducing vascular comorbidities across populations

**DOI:** 10.3389/fcdhc.2025.1695268

**Published:** 2025-10-03

**Authors:** Elise C. Brown, Allan Knox, Kyle Pierce

**Affiliations:** ^1^ Department of Public and Environmental Wellness, Oakland University School of Health Sciences, Rochester, MI, United States; ^2^ Exercise Science Department, California Lutheran University, Thousand Oaks, CA, United States; ^3^ Department of Kinesiology & Health Science, Louisiana State University Shreveport, Shreveport, LA, United States

**Keywords:** resistance training, type 2 diabetes, exercise, vascular complications, physical activity, exercise prescription, diabetes

Type 2 diabetes (T2D) continues to be a significant global health concern (1). The development of this condition is linked to insulin resistance and contributes to micro- and macro-vascular comorbidities such as peripheral neuropathy, retinopathy, and coronary artery disease. Exercise continues to be one of the most effective therapeutic approaches for addressing vascular complications associated with this condition (2, 3). Current research published in the special topic, Exercise and Type 2 Diabetes: Reducing Vascular Comorbidities Across Populations, can directly assist clinicians in exercise prescription and understanding exercise barriers during regular checkups with their patients. This editorial summarizes five studies addressing the improvement of vascular comorbidities in T2D.

Cui et al. conducted a systematic review and network meta-analysis to determine the effectiveness of combined exercise interventions and complementary therapies, such as acupuncture, in improving various cardiometabolic markers in overweight and obese patients with T2D. Combined aerobic and resistance exercise (RE) interventions were the most effective in improving body composition, glycosylated hemoglobin, and Interleukin-6 levels. Physical-mental activities, such as yoga and Tai Chi, appeared to be the most promising interventions for improving blood lipid levels. The authors provided recommendations for clinicians to consider when designing non-pharmacological interventions for their patients, which should be interpreted as maximum viable thresholds. These recommendations included the suggested frequency, duration, and intensity for aerobic exercise, RE, combined aerobic exercise and RE, mind-body movement, and acupuncture. While the authors encouraged clinicians to adapt prescriptions to individuals based on a range of factors such as time constraints, work obligations, and social and financial circumstances, individual recovery from high exercise volume should also be a primary consideration (4). Given that high amounts of exercise can be achieved by following these recommendations’ maximum frequency, duration, and intensity, patients with lower fitness levels may have difficulty recovering from higher volumes of exercise.

Exercise recommendations specific to sensory complication comorbidities associated with T2D have also been proposed. Lopatin et al. discussed how vascular complications in patients with T2D affect the sensory components of balance (*i.e.*, proprioception, vision, and vestibular), and offered exercise prescription recommendations that account for each specific sensory complication. The recommendations emphasized the benefits of Tai Chi for improving balance in people with diabetic peripheral neuropathy (DPN) through increased vascular function and blood flow to the periphery. Additionally, it was suggested that 30–60 minutes of aerobic exercise, 4–6 days a week and RE at least two days a week is beneficial. Specific exercises to improve vestibular function in subjects with diabetic vestibular dysfunction were recommended, including balance movements, goal-directed eye-head exercises, head turns, head-trunk turns, and head-walking turns. The potential risks of weight-bearing activities were addressed for those with DPN, in addition to concerns regarding higher intensities for individuals with more progressed diabetic retinopathy. These exercise recommendations may aid clinicians in determining exercise guidance for their patients, specific to the sensory complications affecting their patients’ balance.

RE prescription can be complex, and there is a need for guidance that is tailored to improving physical function and cardiometabolic health in patients with T2D. The perspective article authored by Brown et al. discussed the necessity of accurate, clear, and standardized definitions for RE movement patterns. The authors aimed to address low RE adherence levels by simplifying the language used to prescribe RE with a specific focus on functional movement patterns. This approach is intended to assist clinicians, scientists, and exercise practitioners in designing and monitoring exercise prescriptions, in addition to removing complexities for those engaging in RE. The article also highlighted the need for medical professionals to become familiar with the potential of RE for clinical populations and encouraged the adoption of the proposed definitions to enhance the safe and effective treatment of patients through RE. The authors proposed a small but potentially impactful method to increase RE participation to ultimately improve the outcomes and daily living standards of T2D patients. The use of the proposed standard definitions can provide the framework required for patient care and surveillance, and improve the reproducibility of methodologies across clinical research, which can be integrated into current exercise guidelines.

The relationship between regular exercise, health checkups and hospitalizations has also been explored. The manuscript by Hamasaki and Yanai investigated the link between periodic health checkups and the risk of hospitalization in patients with T2D in a Japanese cohort. The retrospective study included over two years’ worth of data, which included 1256 patients. The data showed a significant beneficial impact of periodic health checkups on reduced hospitalization risk when adjusting for body composition, social habits, hemodynamics, exercise participation, and metabolic markers. Additionally, traditional body composition and physical activity markers were more favorable in subjects who received periodic health checkups compared to those who did not, which may help interpret the lower hospitalization rates. The data provided by the authors suggested that the impact of periodic health checkups can reduce the severity of T2D to a degree that does not require hospitalization, potentially due to increased clinical oversight. These checkups may provide a platform for consistent, individualized therapy for patients at risk of hospitalization, and could be implemented in this specific population.

While regular exercise decreases the risk of hospitalization in patients with T2D, this population is less physically active (5, 6), possibly due to the greater perceived and physiological effort required during exercise. Huebschmann et al. investigated whether individuals with T2D experience greater physical, physiological, and perceptual effort during low- to moderate-intensity exercise compared to individuals without diabetes. Sex differences were investigated since previous research has shown this effect in women only. More specifically, the researchers measured heart rate and blood lactate, which are parameters used to estimate the degree of physiological stress imposed by physical exercise. They also included a subjective measure of effort, rating of perceived exertion, during treadmill walking at speeds below the aerobic threshold. Although greater physiological markers of effort were associated with having T2D, perceived effort was similar between groups. The authors addressed a crucial barrier to exercise in diabetes management, in that if exercise feels harder physiologically and psychologically, people may be less likely to engage in it regularly. Understanding this struggle to maintain regular physical activity could help design better interventions to promote physical activity and reduce cardiovascular risk in this population.

Regular exercise is effective in improving a range of vascular complications in T2D, and guidance for clinicians on how to prescribe exercise is necessary for providing patients with the necessary tools for the prevention of complications. These exercise prescriptions can be provided during regular checkups, which may further help to reduce the risk of hospitalizations. Understanding of higher levels of effort regarding exercise in T2D may also aid clinicians in having empathetic conversations with patients when discussing exercise barriers.
